# Diabetes knowledge and care practices among adults in rural Bangladesh: a cross-sectional survey

**DOI:** 10.1136/bmjgh-2018-000891

**Published:** 2018-07-23

**Authors:** Edward Fottrell, Naveed Ahmed, Sanjit Kumar Shaha, Hannah Jennings, Abdul Kuddus, Joanna Morrison, Kohenour Akter, Badrun Nahar, Tasmin Nahar, Hassan Haghparast-Bidgoli, A K Azad Khan, Anthony Costello, Kishwar Azad

**Affiliations:** 1 Institute for Global Health, University College London, London, UK; 2 Diabetic Association of Bangladesh, Dhaka, Bangladesh

**Keywords:** epidemiology, prevention strategies, public health, diabetes, community-based survey

## Abstract

**Background:**

Population knowledge of how to prevent, detect and control diabetes is critical to public health initiatives to tackle the disease. We undertook a cross-sectional survey of adults in rural Bangladesh to estimate knowledge and practices related to diabetes.

**Methods:**

In 96 villages in Faridpur district, trained fieldworkers surveyed 12 140 randomly selected men and women aged ≥30. They collected data on sociodemographic status, knowledge of diabetes and history of blood and urine glucose testing. Fasting and 2-hour post-glucose load capillary blood tests ascertained the diabetic status of respondents. Levels of knowledge and practices were analysed by sociodemographic characteristics and diabetic status.

**Results:**

The population showed low levels of diabetes knowledge overall, with only one in three adults able to report any valid causes of the disease. Knowledge of diabetes causes, symptoms, complications, prevention and control was significantly associated with age, education, wealth and employment. Only 14% of respondents reported ever having had a blood glucose test and strong associations with wealth were observed (least poor relative to most poor 2.91 (2.32–3.66)). 78.4% of known diabetics (ie, with a prior diagnosis) reported that they did not monitor their blood glucose levels on at least a monthly basis. However, they had better knowledge of the causes (odds relative to normoglycaemic individuals 1.62 (1.23–2.09)), symptoms (5.17 (3.41–7.82)), complications (5.18 (3.75–7.14)), prevention (4.18 (3.04–5.74)) and control (8.43 (4.83–14.71)).

**Conclusion:**

Knowledge of diabetes among rural adults in Faridpur is extremely poor. Levels of diabetes testing are low and monitoring of blood glucose among known diabetics infrequent. Diabetes prevention and control efforts in this population must include large-scale awareness initiatives which focus not only on high-risk individuals but the whole population.

**Trial registration number:**

ISRCTN41083256; Pre-results.

**Key questions**

**What is already known?**

- Despite high levels of diabetes and intermediate hyperglycaemia in Bangladesh, knowledge and control of the disease is low.
- Knowledge of diabetes prevention, control, consequences and risk factors is significantly associated with education, income and history of disease.
- Population knowledge of how to prevent, detect and control diabetes is critical to public health initiatives to tackle the disease.

**What are the new findings?**

- Only one in three adults in a rural area of Bangladesh are able to report any valid causes of diabetes or methods to prevent it.
- More than three quarters of known diabetics do not monitor their blood glucose on at least a monthly basis.
- Knowledge of diabetes and glucose monitoring practices is associated with sociodemographic parameters of education, wealth, occupation and age.

**What do the new findings imply?**

- Findings indicate low levels of health (diabetes) literacy and inadequate availability and access to information and health and diagnostic services in rural Bangladesh.
- Rural public health strategies that promote knowledge and understanding of diabetes are urgently needed.

## Background

The global prevalence of type 2 diabetes mellitus was estimated to be approximately 9% among adults in 2015, with around 75% of people living with diabetes in low-income and middle-income countries.^1^ Uncontrolled diabetes mellitus is a major cause of chronic morbidity including stroke, renal failure, visual impairment and neuropathy. It has significant impacts on the quality of life and prosperity of individuals, households and communities.^2 3^ Increasing rates of diabetes place a substantial burden on overstretched healthcare services in resource-poor settings that face a triple burden of infectious diseases, injuries and non-communicable diseases.^4^ Underlying the increasing prevalence of diabetes are complex genetic, environmental and lifestyle factors, including infant malnutrition, dietary changes and lack of physical activity.^5–7^

Our large cross-sectional survey in rural Bangladesh in 2016 estimated the prevalence of diabetes to be 8.9% and 11.4% among men and women aged ≥30 years, respectively (Fottrell E, Submiited, 2018). Further, approximately 17% of men and 23% of women were identified to have impaired fasting glucose or impaired glucose tolerance, collectively termed intermediate hyperglycaemia. These estimates are comparable with other estimates.^8–11^ Despite the high levels of diabetes and intermediate hyperglycaemia, awareness and control of the condition is low. In our 2016 survey, we found that only 25% of diabetics were aware of their status, women with diabetes were 37% less likely than men to know that they were diabetic and, even among known diabetics, 75% had suboptimal control of the condition (Fottrell E, Submiited, 2018). The Bangladesh Demographic and Health Survey also found low levels of awareness and control among a diabetic subsample,^8^ though general knowledge of diabetes prevention and control in the population was not investigated.

Reasons for low levels of awareness of one’s diabetic status and control of it include a lack of widespread public health and awareness campaigns, inadequate health services for diagnosing and treating diabetes effectively, people not being able to afford regular healthcare and treatment, and communicable diseases remaining a priority for Bangladeshi public health programmes.^12^ A study by Islam *et al* looking at knowledge of diabetes and glycaemic control among patients with diabetes in urban Dhaka, the capital, found that 46% of patients in the study had good, 38% moderate and 17% poor knowledge on diabetes.^13^ In a separate study in rural Bangladesh, knowledge that diabetes can cause eye disease and can be controlled by regular exercise was found to be higher among men.^14^ Unsurprisingly, knowledge of diabetes prevention, control, consequences and risk factors is significantly associated with higher education, higher monthly income, family history of diabetes and a longer duration of a diagnosis of diabetes.^13 14^

In the current study, we describe diabetes knowledge and care practices among a large rural Bangladeshi population, measured as part of a baseline survey for a cluster randomised controlled trial for diabetes prevention and control led by the Diabetic Association of Bangladesh and University College London.^15^

## Methods

### Study population and sample

The study was conducted in Faridpur district and included 96 rural villages in four upazillas—Nagarkanda, Boalmari, Saltha and Madhukhali—covering a population of approximately 125 000 adults aged ≥30 years. Primary to tertiary levels of healthcare are provided in Faridpur, but distance, long travelling time and a shortage of facilities, trained healthcare providers and medicines are ongoing challenges affecting access and quality of care.

The study population includes male and non-pregnant female permanent residents of the 96 villages aged  ≥30 years. Someone was considered a permanent resident of a village if they normally live in that village. The study team conducted a census of all households and eligible residents to create a sampling frame from which a sample of 143 adults aged ≥30 years in each village was selected using multistage random sampling from a purpose-made sampling frame of all eligible individuals. In the first stage, 143 households with at least one eligible adult resident was selected using probability proportional to size sampling. At the next stage, a single eligible adult was selected for inclusion in the survey using simple random sampling. The sample size was determined by trial requirements described elsewhere^15^ but allows estimation of the true population prevalence of intermediate hyperglycaemia and diabetes mellitus with 99% confidence and an accuracy of between 1% and 2%.

### Data collection

Data were collected by 16 male and 16 female fieldworkers who were recruited locally and received approximately 1 month’s training on survey methods. Male and female fieldworker pairs were supervised by one of four field supervisors who would spend at least half a day observing and verifying data within each pair at least every two days.

Field testing centres were established for the purposes of the study and were at a central, convenient location in each village. All consenting sampled individuals were requested to attend these centres on the morning of a specified day following an overnight fast for a range of physical measurements, including blood glucose. Blood glucose was measured using the One Touch Ultra Glucometer (Lifescan, Milpitas, California, USA) in whole blood obtained by finger prick from capillaries in the middle or ring finger after an overnight fast. All individuals then received a 75 g glucose load dissolved in 250 mL of water and had a repeat capillary blood test within 120 min (±5 min) post ingestion to determine glucose tolerance status and differentiate between individuals with intermediate hyperglycaemia and those with diabetes according to WHO criteria^16^ or a prior medical diagnosis of diabetes.

Sociodemographic, lifestyle and behavioural data of all consenting individuals were collected through interview using a structured survey instrument. Questionnaire data were gathered using Samsung Galaxy Grand Prime smartphones using Open Data Kit Collect software. Collection of questionnaire data took place at the respondent’s home before or after the physical measurements or at the testing centre at the time of physical measurement. Data were uploaded from mobile phones to the supervisors’ laptop every two days and then transferred to a central database at the Faridpur field office for further data checks and quality control before being transferred on a weekly basis to the main project office in Dhaka.

Knowledge of diabetes was measured among all participants by asking a series of questions on (a) whether they were able to report any valid causes of diabetes, (b) whether they were able to report any valid symptoms of diabetes, (c) whether they were able to report any valid complications of diabetes, (d) whether they were able to report any valid ways to prevent diabetes and (e) whether they were able to report any valid ways to control diabetes if one has it. All of these questions were open-ended and unprompted, with fieldworkers ticking all valid responses from a checklist developed by the study team following review of the literature and discussion with diabetes specialists at the Bangladesh Institute of Research and Rehabilitation for Diabetes, Endocrine and Metabolic Disorders in Dhaka. The checklists were piloted in the study area and are available as online supplementary table 1. In addition, we gathered self-reported data on whether respondents had ever had their urine or blood tested for sugar. Finally, known diabetics were asked how often they had had their blood sugar checked since diagnosis.

10.1136/bmjgh-2018-000891.supp1 Supplementary file 1

### Analyses

Descriptive analysis summarised levels of knowledge and practices by study population characteristics and diabetic status categorised as normoglycaemia (fasting glucose <6.1 mmol/L), intermediate hyperglycaemia (including impaired fasting glucose (fasting glucose ≥6.1 mmol/L to <7.0 mmol/L and 2-hour post ingestion of 75 g glucose load blood glucose <7.8 mmol/L) and impaired glucose tolerance (fasting glucose ≥6.1 mmol/L to <7.0 mmol/L and 2-hour post ingestion of 75 g glucose load blood glucose ≥7.8 mmol/L and <11.1 mmol/L)), unknown diabetic (diabetic (fasting glucose ≥7.0 mmol/L or 2-hour post ingestion of 75 g glucose load blood glucose ≥11.1 mmol/L) who did not know of their diabetic status prior to our survey) and known diabetic (diabetic with who had previously been informed of their diabetic status). Households were categorised into five socioeconomic quintiles using a wealth index derived from principal components analysis of household’s ownership of assets, housing characteristics, sanitation facilities and land ownership.^17^ Associations between diabetes knowledge and practices with sociodemographic factors and diabetic status were assessed using crude and multivariate logistic regression. We reason that all independent variables may bias our results and so all are included in the multivariate models. Analyses stratified by sex were conducted on suggestion from peer reviewers. All analyses were carried out in Stata V.13 and adjusted for the clustered and stratified survey design and weighted to account for the unequal probability of selection of a fixed number of individuals within villages of unequal size using the ‘svy’ command in STATA.

Participation in the surveys was voluntary and informed consent was obtained from all participants before any data were collected.

## Results

### Response rate and study population

Using the sampling frame, we developed a target sample list of 13 684 individuals. Note this number is slightly lower than the expected target of 143 individuals in each of 96 villages (13 728 individuals) as two villages only had 128 and 114 eligible individuals living in separate households, respectively.

Survey data were collected from a total of 12 140 individuals (5684, 46.8% male; 6456, 53.2% female (unweighed proportions)) out of a target 13 684 between January and March 2016, representing an overall response rate of 88.7%. Using sampling frame data, it was possible to explore age and sex bias in response rates. Non-responders were younger (mean 46.5 years (SD 14.8) vs 47.7 years (SD 13.8), p=0.003) and more likely to be male than female (15.7% vs 7.0%, p<0.001). Ninety-three individuals (0.8%) had missing data on diabetic status and two (0.02%) had missing data on occupation and so these individuals are not included in the multivariate analysis.

Study population characteristics are presented in table 1 and show that approximately 90% of the population were currently married and Muslim and overall the population had low levels of education and literacy. More than half of the population (predominately the females) had no paid employment. Approximately 11% of the population were diabetic, although only 24% of these were aware of their diabetic status.

**Table 1:** Crude associations between study population characteristics and ability to correctly report valid answers within five domains of diabetes knowledge

		Total	Proportion able to report valid answers within each of the following domains of knowledge of diabetes									
			Causes		Symptoms		Complications		Prevention		Control	
		N (%)	%	OR (95% CI)	%	OR (95% CI)	%	OR (95% CI)	%	OR (95% CI)	%	OR (95% CI)
Sex	Male	5684 (47.0)	35.4		56.5		29.3		39.7		68.5	
	Female	6456 (53.0)	31.8	0.85 (0.74 to 0.97)	53.5	0.89 (0.80 to 0.98)	25.5	0.82 (0.72 to 0.95)	35.5	0.83 (0.75 to 0.93)	63.6	0.81 (0.72 to 0.90)
Age (years)	30–39	4108 (33.6)	36.8		59.6		29.2		41.8		69.9	
	40–49	3051 (25.2)	35.4	0.94 (0.85 to 1.04)	57.8	0.93 (0.84 to 1.02)	28.6	0.97 (0.88 to 1.07)	39.9	0.92 (0.84 to 1.01)	69.0	0.96 (0.87 to 1.06)
	50–59	2293 (19.0)	33.5	0.86 (0.77 to 0.97)	56.0	0.86 (0.77 to 0.97)	28.6	0.97 (0.85 to 1.11)	36.4	0.80 (0.71 to 0.90)	67.4	0.89 (0.78 to 1.02)
	60–69	1917 (15.9)	29.4	0.71 (0.63 to 0.82)	46.8	0.60 (0.53 to 0.68)	24.4	0.78 (0.67 to 0.91)	31.2	0.63 (0.55 to 0.73)	57.9	0.59 (0.52 to 0.68)
	70+	771 (6.3)	18.5	0.39 (0.30 to 0.51)	34.7	0.36 (0.29 to 0.45)	15.2	0.43 (0.34 to 0.56)	24.2	0.44 (0.36 to 0.56)	48.4	0.40 (0.33 to 0.50)
Currently married	No	1508 (12.2)	25.2		44.6		20.6		30.1		58.1	
	Yes	10 632 (87.8)	34.7	1.58 (1.34 to 1.86)	56.3	1.60 (1.39 to 1.84)	28.2	1.52 (1.30 to 1.77)	38.5	1.46 (1.25 to 1.70)	67.0	1.46 (1.27 to 1.69)
Education	No formal	6057 (49.5)	25.7		43.8		18.8		26.6		55.9	
	Incomplete primary	2777 (23.3)	37.1	1.71 (1.46 to 2.00)	61.8	2.07 (1.80 to 2.38)	28.7	1.74 (1.47 to 2.07)	40.8	1.90 (1.63 to 2.21)	73.1	2.14 (1.83 to 2.50)
	Completed primary or above	3306 (27.2)	44.7	2.34 (2.02 to 2.71)	69.0	2.85 (2.47 to 3.30)	41.5	3.07 (2.61 to 3.61)	54.4	3.29 (2.85 to 3.80)	77.9	2.78 (2.35 to 3.27)
Literate	Illiterate	7475 (61.4)	27.0		47.0		20.9		28.9		59.5	
	Literate	4665 (38.6)	43.8	2.11 (1.87 to 2.39)	67.4	2.34 (2.08 to 2.63)	37.5	2.27 (1.98 to 2.60)	51.1	2.58 (2.27 to 2.92)	76.1	2.16 (1.91 to 2.44)
Occupation*	Unemployed	6703 (55.1)	31.3		52.6		25.2		35.2		63.1	
	Manual	4034 (33.3)	32.9	1.07 (0.94 to 1.23)	53.8	1.05 (0.94 to 1.17)	25.9	1.04 (0.91 to 1.18)	35.8	1.03 (0.91 to 1.15)	66.7	1.17 (1.04 to 1.32)
	Professional	1401 (11.6)	45.7	1.84 (1.56 to 2.17)	68.7	1.97 (1.69 to 2.31)	41.0	2.06 (1.74 to 2.45)	53.5	2.12 (1.81 to 2.48)	76.9	1.95 (1.63 to 2.32)
Wealth	Most poor	2431 (19.7)	23.0		42.3		18.8		25.5		55.2	
	2	2445 (20.4)	28.5	1.33 (1.12 to 1.59)	46.9	1.20 (1.03 to 1.41)	20.4	1.10 (0.92 to 1.32)	26.6	1.06 (0.90 to 1.25)	53.9	0.95 (0.76 to 1.18)
	3	2441 (20.2)	29.9	1.42 (1.20 to 1.69)	53.6	1.57 (1.31 to 1.89)	24.0	1.36 (1.12 to 1.67)	33.2	1.45 (1.20 to 1.76)	64.9	1.50 (1.22 to 1.83)
	4	2403 (19.6)	35.6	1.85 (1.51 to 2.26)	59.5	2.01 (1.64 to 2.45)	28.7	1.74 (1.37 to 2.20)	41.4	2.07 (1.65 to 2.58)	73.2	2.21 (1.82 to 2.67)
	Least poor	2420 (20.1)	50.3	3.38 (2.69 to 4.26)	72.0	3.50 (2.75 to 4.46)	44.3	3.43 (2.71 to 4.34)	60.9	4.55 (3.62 to 5.73)	82.5	3.81 (2.93 to 4.95)
Religion	Other	1140 (9.2)	33.2		55.0		31.9		44.4		70.0	
	Muslim	11 000 (90.8)	33.5	1.02 (0.71 to 1.45)	54.8	0.99 (0.73 to 1.34)	26.8	0.78 (0.57 to 1.08)	36.8	0.73 (0.53 to 1.00)	65.5	0.81 (0.55 to 1.21)
Diabetic status*	Normoglycaemic	8364 (69.2)	33.0		54.6		26.1		36.3		65.4	
	Intermediate hyperglycaemia	2455 (20.5)	33.0	1.00 (0.87 to 1.15)	51.9	0.90 (0.78 to 1.03)	25.6	0.98 (0.85 to 1.12)	37.1	1.03 (0.91 to 1.17)	64.4	0.96 (0.84 to 1.08)
	Unknown diabetic	918 (7.7)	33.8	1.03 (0.86 to 1.24)	53.9	0.97 (0.81 to 1.17)	28.4	1.12 (0.91 to 1.39)	37.7	1.06 (0.87 to 1.29)	63.6	0.92 (0.76 to 1.13)
	Known diabetic	310 (2.6)	49.4	1.98 (1.53 to 2.56)	87.1	5.63 (3.72 to 8.54)	68.4	6.12 (4.58 to 8.18)	73.9	4.96 (3.69 to 6.69)	94.5	9.17 (5.24 to 16.05)
Total		12 140 (100.0)	33.5		54.9		27.3		37.5		65.9	

Proportions are cluster means and odds ratios and 95% CIs are adjusted for the stratified, clustered survey design.

*Missing diabetic status for 93 individuals; missing occupation data for two individuals.

### Knowledge

Overall, 33.5% of the population were able to report any valid causes of diabetes, with approximately 55% being aware of any symptoms of diabetes and approximately 27% able to report ways to prevent the disease (table 1). Only 37.5% of respondents were aware of the specific complications of diabetes. However, two-thirds of respondents were able to report at least one medical intervention or change in behaviour that could be used to control diabetes if one has the condition.

All observed differences in knowledge between men and women became non-significant on adjustment for other sociodemographic and diabetic status factors (table 2). Decreasing knowledge with age and increasing knowledge with education level was observed. Positive effects of literacy in the unadjusted, crude analysis only remained significant for ability to correctly report causes of diabetes when adjusted for other variables.

**Table 2:** Adjusted ORs showing associations between study population characteristics and ability to correctly report valid answers within five domains of diabetes knowledge

		Ability to report valid answers within each of the following domains of knowledge of diabetes				
		Causes	Symptoms	Complications	Prevention	Control
		AOR 95% CI	AOR 95% CI	AOR 95% CI	AOR 95% CI	AOR 95% CI
Sex	Male					
	Female	1.02 (0.78 to 1.32)	1.13 (0.93 to 1.37)	1.05 (0.80 to 1.37)	0.99 (0.78 to 1.24)	0.96 (0.78 to 1.19)
Age (years)	30–39					
	40–49	1.02 (0.91 to 1.13)	1.03 (0.93 to 1.15)	1.08 (0.97 to 1.21)	1.01 (0.90 to 1.13)	1.03 (0.92 to 1.15)
	50–59	1.00 (0.88 to 1.13)	1.04 (0.91 to 1.19)	1.15 (0.99 to 1.34)	0.91 (0.78 to 1.05)	1.00 (0.87 to 1.15)
	60–69	0.84 (0.73 to 0.97)	0.73 (0.63 to 0.84)	0.94 (0.79 to 1.11)	0.71 (0.59 to 0.84)	0.66 (0.56 to 0.78)
	70+	0.45 (0.34 to 0.60)	0.42 (0.33 to 0.53)	0.51 (0.37 to 0.69)	0.47 (0.35 to 0.62)	0.41 (0.33 to 0.52)
Currently married	No					
	Yes	1.04 (0.87 to 1.25)	0.99 (0.85 to 1.16)	1.01 (0.85 to 1.20)	0.87 (0.73 to 1.04)	0.84 (0.71 to 1.00)
Education	No formal					
	Incomplete primary	1.22 (0.97 to 1.55)	1.67 (1.36 to 2.04)	1.61 (1.27 to 2.05)	1.47 (1.18 to 1.82)	1.91 (1.54 to 2.37)
	Completed at least primary	1.16 (0.84 to 1.60)	1.77 (1.34 to 2.33)	2.40 (1.78 to 3.23)	1.80 (1.36 to 2.39)	2.12 (1.56 to 2.88)
Literate	Illiterate					
	Literate	1.40 (1.07 to 1.83)	1.12 (0.88 to 1.42)	0.91 (0.71 to 1.18)	1.16 (0.89 to 1.50)	0.84 (0.66 to 1.08)
Occupation	Unemployed					
	Manual	1.12 (0.88 to 1.42)	1.17 (0.98 to 1.40)	1.15 (0.88 to 1.50)	1.11 (0.89 to 1.40)	1.18 (0.96 to 1.45)
	Professional	1.34 (1.08 to 1.66)	1.49 (1.21 to 1.83)	1.44 (1.13 to 1.85)	1.38 (1.11 to 1.71)	1.29 (1.02 to 1.64)
Wealth	Most poor					
	2	1.27 (1.07 to 1.52)	1.14 (0.97 to 1.33)	1.03 (0.85 to 1.24)	1.01 (0.85 to 1.19)	0.90 (0.72 to 1.12)
	3	1.27 (1.07 to 1.52)	1.41 (1.17 to 1.71)	1.20 (0.98 to 1.47)	1.29 (1.06 to 1.58)	1.38 (1.13 to 1.70)
	4	1.60 (1.29 to 1.98)	1.69 (1.37 to 2.09)	1.39 (1.08 to 1.78)	1.71 (1.35 to 2.16)	1.93 (1.59 to 2.35)
	Least poor	2.70 (2.14 to 3.41)	2.62 (2.01 to 3.41)	2.31 (1.79 to 2.99)	3.32 (2.55 to 4.31)	3.06 (2.33 to 4.02)
Religion	Other					
	Muslim	1.18 (0.82 to 1.70)	1.17 (0.85 to 1.60)	0.94 (0.66 to 1.33)	0.86 (0.63 to 1.18)	0.93 (0.62 to 1.40)
Diabetic status	Normoglycaemic					
	Intermediate hyperglycaemia	1.03 (0.89 to 1.18)	0.92 (0.79 to 1.07)	0.99 (0.86 to 1.14)	1.06 (0.93 to 1.20)	0.99 (0.87 to 1.14)
	Unknown diabetic	1.01 (0.82 to 1.23)	0.96 (0.79 to 1.18)	1.09 (0.87 to 1.36)	1.01 (0.82 to 1.25)	0.93 (0.75 to 1.14)
	Known diabetic	1.61 (1.23 to 2.09)	5.17 (3.41 to 7.82)	5.18 (3.75 to 7.14)	4.18 (3.04 to 5.74)	8.43 (4.83 to 14.71)

Results are adjusted for all covariates and for the stratified, clustered survey design.

n for multivariate analysis=12 045 due to missing occupation or diabetic status data for 95 individuals.

Knowledge of the causes, symptoms, complications, prevention and control of diabetes was greater among professionally employed individuals relative to the unemployed group, even after controlling for other sociodemographic and diabetic status variables. Similarly, a dose–response relationship between household wealth and diabetes knowledge was observed, with increasing knowledge among individuals from higher (less poor) wealth quintiles.

With regards to diabetic status, it is notable that there were no significant differences in knowledge between normoglycaemic individuals and those with intermediate hyperglycaemia or unknown diabetes. Known diabetics had significantly improved knowledge of diabetes, although levels remained low in absolute terms, with less than half knowing the cause of their disease.

The top ranking (frequency of 10% or more) reported causes, symptoms, complications, prevention and control strategies reported by respondents are summarised in Box 1.

Box 1 **Top ranking reported diabetes causes, symptoms, complications, prevention and control strategies among 12 140 adults in rural Bangladesh**

**Causes** *(able to report any valid causes=33.5%)*

Excessive sugar consumption (88.1%)

Lack of physical activity (29.3%)

Dietary factors, such as over consumption of fats and/or carbohydrate (21.8%)

**Symptoms** (a*ble to report any valid symptoms=54.9%)*

Excessive/frequent urination (94.7%)

Fatigue (18.9%)

Cuts or wounds that are slow to heal (13.9%)

Unexpected weight loss (11.6%)

Passage of sugar in urine (sometimes described as ants being drawn to urine) (10.3%)

**Complications** (*able to report any valid complications=27.3%)*

Renal problems, including frequent urination (49.5%)

Cuts, wounds and skin infections being slow to heal/not healing (36.4%)

Vision/eye problems (29.1%)

**Prevention** *(able to report any valid prevention strategies=37.5%)*

Reduced sugar consumption in diet (69.9%)

Increased physical activity (58.8%)

Control of other dietary factors (eg, reduced fat and carbohydrate consumption) (53.2%)

**Control** *(able to report any valid control strategies=65.9%)*

Dietary practices (eg, reduced fat and carbohydrate consumption) (56.6%)

Increased physical activity (53.9%)

Reduced sugar consumption (52.9%)

Pharmaceutical medicines (32.2%)

Percentages represent the frequency of reports among those able to report at least one valid item.

### Care practices

Only 14% of respondents reported ever having a blood glucose test, and fewer (5.2%) reported having a urine sugar test, with no differences observed between men and women (table 3). Increasing likelihood of blood or urine sugar testing was observed among older and more educated respondents. Observed crude associations between literacy and occupation became non-significant in multivariate analysis. Strong associations between wealth and ever having had a blood glucose test were observed, although even in the wealthiest quintile less than one-third of respondents had ever had a blood glucose test. The association between wealth and urine testing is less strong, with a significant association only being observed in the least poor group relative to the poorest.

**Table 3:** Frequency, crude and adjusted ORs and 95% CIs for blood glucose testing and urine glucose testing by sociodemographic characteristic

		Total		Ever blood glucose test			Ever urine glucose test		
		n	%	%	OR 95% CI	AOR 95% CI	%	OR 95% CI	AOR 95% CI
Sex	Male	5684	47.0	13.9			5.3		
	Female	6456	53.0	14.0	1.01 (0.90 to 1.13)	0.88 (0.68 to 1.15)	5.0	0.94 (0.79 to 1.11)	0.78 (0.55 to 1.11)
Age (years)	30–39	4108	33.6	9.8			3.5		
	40–49	3051	25.2	13.0	1.37 (1.20 to 1.57)	1.55 (1.33 to 1.81)	4.1	1.18 (0.94 to 1.49)	1.11 (0.86 to 1.45)
	50–59	2293	19.0	17.9	2.02 (1.72 to 2.37)	2.57 (2.13 to 3.08)	6.8	1.99 (1.57 to 2.52)	1.93 (1.45 to 2.56)
	60–69	1917	15.9	18.7	2.13 (1.80 to 2.51)	2.59 (2.09 to 3.22)	7.9	2.34 (1.88 to 2.90)	2.12 (1.60 to 2.81)
	70+	771	6.3	16.5	1.82 (1.46 to 2.27)	1.80 (1.32 to 2.45)	6.5	1.89 (1.35 to 2.66)	1.54 (0.99 to 2.41)
Currently married	No	1508	12.2	16.5			5.9		
	Yes	10 632	87.8	13.6	0.80 (0.69 to 0.94)	0.76 (0.62 to 0.93)	5.1	0.85 (0.67 to 1.07)	0.88 (0.63 to 1.22)
Education	No formal	6057	49.5	9.4			3.6		
	Incomplete primary	2777	23.3	12.8	1.42 (1.21 to 1.66)	1.61 (1.26 to 2.05)	4.8	1.35 (1.07 to 1.71)	1.69 (1.21 to 2.36)
	Completed at least primary	3306	27.2	23.2	2.90 (2.47 to 3.41)	2.50 (1.86 to 3.35)	8.4	2.46 (1.97 to 3.07)	2.20 (1.39 to 3.48)
Literate	Illiterate	7475	61.4	10.0			3.8		
	Literate	4665	38.6	20.4	2.31 (2.03 to 2.63)	1.15 (0.90 to 1.47)	7.3	1.99 (1.66 to 2.39)	0.98 (0.67 to 1.45)
Occupation	Unemployed	6703	55.1	15.3			5.7		
	Manual	4034	33.3	9.1	0.55 (0.47 to 0.65)	0.58 (0.43 to 0.78)	3.1	0.53 (0.43 to 0.66)	0.52 (0.36 to 0.75)
	Professional	1401	11.6	21.7	1.54 (1.31 to 1.80)	0.87 (0.64 to 1.19)	8.4	1.51 (1.17 to 1.95)	0.89 (0.60 to 1.32)
Wealth	Most poor	2431	19.7	7.8			3.3		
	2	2445	20.4	7.4	0.94 (0.71 to 1.24)	0.84 (0.62 to 1.14)	3.4	1.04 (0.70 to 1.54)	0.97 (0.66 to 1.42)
	3	2441	20.2	11.1	1.47 (1.18 to 1.82)	1.32 (1.05 to 1.64)	4.4	1.37 (0.98 to 1.92)	1.21 (0.88 to 1.66)
	4	2403	19.6	14.4	1.97 (1.59 to 2.45)	1.60 (1.27 to 2.01)	4.8	1.50 (1.10 to 2.04)	1.16 (0.86 to 1.57)
	Least poor	2420	20.1	29.2	4.86 (3.95 to 5.98)	2.91 (2.32 to 3.66)	9.9	3.23 (2.36 to 4.42)	1.53 (1.09 to 2.13)
Religion	Other	1140	9.2	20.3			9.0		
	Muslim	11 000	90.8	13.3	0.60 (0.47 to 0.77)	0.78 (0.60 to 1.02)	4.8	0.51 (0.34 to 0.77)	0.64 (0.42 to 0.98)
Diabetic status	Normoglycaemic	8364	69.2	11.0			3.4		
	Intermediate hyperglycaemia	2455	20.5	12.4	1.15 (0.97 to 1.35)	1.03 (0.88 to 1.21)	4.0	1.17 (0.92 to 1.48)	1.06 (0.84 to 1.34)
	Unknown diabetic	918	7.7	17.3	1.70 (1.38 to 2.09)	1.36 (1.09 to 1.69)	6.0	1.79 (1.31 to 2.43)	1.50 (1.10 to 2.05)
	Known diabetic	310	2.6	98.0	400.02 (168.77 to 948.13)	348.01 (141.51 to 855.86)	59.6	41.50 (30.81 to 55.90)	28.85 (20.87 to 39.89)
Total		12 140	100	14.0			5.2		

Proportions are cluster means. All ORs and 95% CIs are adjusted for the stratified, clustered survey design. Adjusted ORs are adjusted for all covariates.

n for multivariate analysis=12 045 due to missing occupation or diabetic status data for 95 individuals.

Unsurprisingly, almost all known diabetics reported ever having received a blood glucose test, and they were far more likely than any other group to have had either their blood or urine tested. Interestingly, unknown diabetics had significantly higher odds of blood and urine glucose testing relative to normoglycaemic individuals.

Among diabetic individuals who reported being aware of their diabetic status for at least 1 month prior to our survey (301/310, 97%), 174 (59.5%) reported that they had checked their blood glucose levels less often than once per month in the past six months and 60 (18.9%) reported that they had never checked their blood glucose within the same time period. We found no significant associations between at least monthly blood glucose monitoring and any of our sociodemographic parameters in a multivariate analysis (data not shown).

### Gender effects

Despite some differences in associations reaching statistical significance, there were no major variability in the magnitude and direction of associations between socioeconomic parameters and knowledge of diabetes (online supplementary tables 2 to 5). In terms of blood or urine glucose monitoring, most associations with socioeconomic parameters were of a similar size and magnitude not to lead to different conclusions or public health action (online supplementary tables 6 and 7). However, observed differences in the effect of occupation, whereby employed men were significantly less likely to have ever had a blood glucose test, were not observed among women. Among women, the odds of unknown diabetics ever having blood or urine glucose tests was significantly higher compared with normoglycaemic individuals, whereas unknown diabetic men were no more likely than normoglycaemic individuals to have had the same tests.

## Discussion

Our population-based survey of adults aged ≥ 30 years in rural Bangladesh shows extremely low levels of knowledge of the causes, symptoms, complications and modes of prevention and control of diabetes. Reflecting this general lack of knowledge, population prevalence of testing for blood or urine sugar is remarkably low. Although important differences can be observed across socioeconomic characteristics, the low levels of knowledge and testing is universal, with approximately half of even the most ‘advantaged’ groups (in terms of education, wealth quintile, literacy) unable to correctly report the causes of diabetes and approximately one-third unable to correctly report the symptoms of the disease. Given the high prevalence of diabetes in this population, and the looming threat of a greater burden of disease from the high prevalence of intermediate hyperglycaemia and major risk factors (Fottrel, submitted, 2018), this lack of knowledge presents a major public health threat.

The large, exclusively rural, random sample population and high response rates are major strengths of this study. Our questionnaire gathered data on unprompted knowledge of diabetes, which more realistically tests our respondents’ ability to spontaneously recall and report their understanding of diabetes than relying on recognition of cases, symptoms, prevention and control when presented with a list of options or yes/no response categories. This approach is also more likely to reduce reporting bias.

Our study has several limitations however. First, there appears to have been some response bias in terms of age, with more male and younger non-responders. The reasons for these differences probably relate to competing work responsibilities among younger men. This bias means that our findings may under-represent younger men and so potentially underestimate the knowledge of diabetes overall. Second, although homogeneity in rural Bangladeshi populations has been noted by others,^18^ extrapolation of our findings to other rural areas must be done with caution and our findings are likely to be different from those from an urban or mixed population. We also only included adults aged ≥30 years in our survey. This may have increased the proportion of individuals with diabetes in our sample as age is strongly correlated with the disease. Further, our age restriction may have resulted in a population with lower levels of education and literacy than the population average, especially given considerable progress in female education in particular in Bangladesh in recent years.^8^ Finally, the list of causes, symptoms, complications and methods of prevention and control in our checklists is not exhaustive but rather designed to capture the most likely responses as agreed with experts in Dhaka. This was a pragmatic approach to conducting a knowledge survey in such a large population and, while it is possible that some respondents will have reported other valid responses that would have simply been coded as ‘other’ and therefore not counted as a valid response in our analysis, we expect this was not common and will not have changed our overall assessment of knowledge or our conclusions.

Similar to other studies,^13 19^ knowledge of diabetes and uptake of blood or urine glucose testing was significantly higher among known diabetics, although these represent less than one quarter of diabetics in this population. This finding is not surprising and we have previously reported that most known diabetics do receive at least some treatment or advice for their condition, although for many blood sugar remains poorly controlled (Fottrel, submitted, 2018). While there are no significant differences in knowledge of causes, symptoms, complications, prevention or control of diabetes among unknown diabetics compared with those with normal blood sugar, the fact that female unknown diabetics were significantly more likely to have ever had their blood or urine tested for glucose is notable (online supplementary table 7). Blood or urine glucose testing as part of antenatal care services would not explain why unknown diabetic women were more likely to have been tested than their normoglycaemic counterparts. Rather, these women may have interacted with health service providers at some other point, possibly as a consequence of manifestations of diabetes that they had experienced and the fact that they remain unaware of their diabetic status is concerning.

The other apparent gender difference relates to the effect of occupation, whereby employment appears to increase the likelihood of blood glucose testing among women but reduces it among men, even when controlling for other factors such as education. Explanations for this require further investigation, including qualitative methods, but may include differing opportunities for interaction with healthcare and diagnostic services between occupied men and women. Although women are much less likely to have paid occupations than men, when they do they are more likely to be professional occupations rather than manual occupations compared with men. We know from our qualitative studies in Faridpur that diabetic testing is perceived as time consuming, and so manual labourers, often on a daily wage, risk losing a whole day’s pay if they go for testing. Since employed women are more likely to be in professional occupations, they may have more opportunity to take time off for health reasons.

Among those with known diabetes, the vast majority had not checked their blood glucose levels on at least a monthly basis within the 6 months prior to our survey. Blood glucose testing services are available locally in Faridpur, although the cost of testing and the unreliable availability of glucometer testing strips (essential single-use components of testing) may act as barriers to uptake of these services. Low levels of blood glucose monitoring were also reported by Islam *et al*. They also observed that patterns of blood glucose monitoring did not differ across sociodemographic strata, although in contrast to our results, wealth correlated with increased frequency of monitoring.^14^ The exact role of blood glucose monitoring for adults with type 2 diabetes who are not on insulin or other medication is unclear, although monitoring does improve quality of life and supports self-management of the disease.^9 20^ Further investigation is needed to better understand the reasons for low levels of monitoring and the role it might have in rural Bangladesh.

Our study adds to the sparse literature on knowledge, awareness and practices related to diabetes in rural Bangladesh. Islam *et al* also observed low levels of knowledge of diabetes and its risk factors among adults in rural Narail district^13^ but found differences in knowledge between males and females,^14 21^ which were generally not observed in our population. Studies in more urban populations and health facility-recruited populations^18 19^ had strong selection bias unlike our findings from our rural, population-based random sample.

Knowledge and ‘health literacy’ is critical in the epidemiological transition of disease and the prevention and detection of diabetes in resource-poor settings.^14 19 21^ Increasing diabetes knowledge and testing observed in more educationally and wealth advantaged groups in our study is perhaps not surprising and supports other findings in both high-income and low-income settings.^14 19 22–24^ Health inequalities are well described in Bangladesh^25 26^ and the majority of the predicted rising burden of diabetes in Bangladesh is expected to occur in the low and middle socioeconomic groups where, as we have shown, knowledge of diabetes is poor and opportunities and ability to act to prevent and control the disease may be limited.^18 19^

Current health services and preventative strategies in Bangladesh are inadequately prepared to address the challenges of high disease prevalence, looming increases in prevalence and low levels of knowledge, particularly in rural areas.^18 21 27^ In line with recommendations of the Global Strategy for the Prevention and Control of Non Communicable Diseases, there is a need for large-scale awareness intervention programmes that target not only high-risk individuals, but whole populations.^28 29^ Raising knowledge and awareness of diabetes in the wider population is necessary, not least because previous studies have shown that, in the absence of universal health coverage and access to professional services, the most common source of information on prevention and care seeking for diabetes are family members, friends and neighbours.^21^ The fact that we have observed even a small degree of knowledge within our study population may therefore be seen as a positive base on which to build and increase the spread of knowledge. Mass media health promotion campaigns and opportunities created by the widespread ownership of mobile phones may create opportunities for this. It is important, however, that any such strategies are tailored to the context and literacy of adult rural Bangladeshi populations and inclusive of those with lower education and wealth. Lessons may also be learnt from recent population health gains in Bangladesh, such as progress in maternal and child health, and group and individual knowledge, awareness and behaviour change interventions that have shown success in relation to other health outcomes. There is a need for any such innovations and interventions to be robustly evaluated and evidence-based before scale-up.

## Conclusion

Our cross-sectional survey in a large rural population shows that knowledge on the causes, symptoms, consequences, prevention and control of diabetes is limited in rural Bangladesh. A minority of individuals with diabetes are aware of their status and even then do not appear to monitor their blood glucose levels on a regular basis. These findings may be reflective of low levels of health literacy and inadequate availability and access to health services and information in rural areas. Community-based interventions that promote knowledge and understanding of diabetes are needed and these should capitalise on existing knowledge and prevailing lay networks of health information exchange that may already exist.
