# Supplementary material for: Diabetes knowledge and care practices among adults in rural Bangladesh: a cross-sectional survey
Source: BMJ Glob Health. 2018 Jul 23;3(4):e000891. doi: 10.1136/bmjgh-2018-000891 (PMC6058170; doi:10.1136/bmjgh-2018-000891)
Supplement: Supplementary file 1 [file bmjgh-2018-000891supp001.pdf]

Supplementary Table 1. Translation of the response checklists for causes, symptoms and complications of diabetes to assess population awareness of the disease (original version was in Bangla). Items marked with * indicate a valid response.

Causes of diabetes	Hereditary*
	Diet: excessive sugar/sweets*
	Diet: overeating (e.g. fat, carbohydrate)*
	Diet: other/non-specific*
	Overweight/obesity*
	Lack of exercise/Lack of physical work*
	Smoking*
	Advanced age*
	Other reason not listed above
	Don’t know
Symptoms of diabetes	Excessive thirst*
	Excessive/frequent urination*
	Fatigue*
	Unexpected weight loss*
	Cuts/wounds that heal slowly*
	Blurred vision*
	Fungal infections (e.g vaginal thrush/oral candidiasis)*
	Passage of sugar with urine (Ants are drawn to the urine)*
	Itching*
	Increased appetite (frequent hunger)*
	Other reason not listed above
	Don’t know
Complications of diabetes	Eye problems/blurred vision/loss of vision/blindness*
	Kidney problems (frequent urination, ankle/leg swelling, nausea/vomiting, weakness/fatigue)*
	Heart problems (chest pains, angina, heart attack)*
	Cerebrovascular Stroke*
	Nerve damage (pain, tingling, loss of feeling)*
	Incontinence of urine*
	Impotence*
	Gangrene/Amputation/loss of limbs*
	Cuts/blisters/ulcers/skin infections healing slowly or not healing properly*
	Pregnancy complications*
	Disability (non-specific)*
	Gingivitis/tooth infections*
	TB*
	Other reason not listed above
	Don’t know
Prevention/Control of diabetes	Pharmaceutical drugs*
	Insulin*
	Diet: Reduce carbohydrate intake*
	Diet:Reduce sugar intake*
	Diet:Reduce fat intake*
	Diet:Other/general dietary change*
	Reduce weight*
	Stop smoking*
	Start or do more exercise*

	Reduce tension/stress*
	Cannot prevent
	Cannot control
	God’s will
	Regular visits to the doctor/check-ups* (*valid for control only)*
	Other reason not listed above
	Don’t know

Supplementary Table 2 Crude associations between MALE study population characteristics and ability to correctly report valid answers within five domains of diabetes knowledge. Proportions are cluster means and odds ratios and 95% confidence intervals are adjusted for the stratified, clustered survey design.

			Proportion able to report valid answers within each of the following domains of knowledge of diabetes:									
		Total	Causes		Symptoms		Complications		Prevention		Control	
		N (%)	%	OR (95% CI)	%	OR (95% CI)	%	OR (95% CI)	%	OR (95% CI)	%	OR (95% CI)
Age (years)	30-39	1732 (30.0)	39.5		61.3		30.4		44.2		73.4	
	40-49	1376 (24.3)	36.1	0.87 (0.74 - 1.02)	57.7	0.86 (0.74 - 1.00)	30.4	1.00 (0.85 - 1.18)	40.6	0.86 (0.75 - 0.99)	70.1	0.85 (0.73 - 0.99)
	50-59	1135 (20.3)	35.4	0.84 (0.71 - 1.00)	57.3	0.85 (0.72 - 1.00)	31.5	1.05 (0.88 - 1.25)	38.8	0.80 (0.68 - 0.94)	70.0	0.85 (0.71 - 1.00)
	60-69	1039 (18.4)	32.4	0.73 (0.62 - 0.87)	51.7	0.68 (0.56 - 0.81)	27.0	0.84 (0.68 - 1.04)	35.4	0.69 (0.58 - 0.82)	62.2	0.60 (0.49 - 0.72)
	70+	402 (7.0)	24.1	0.49 (0.35 - 0.67)	41.7	0.45 (0.35 - 0.59)	20.2	0.58 (0.43 - 0.79)	32.2	0.60 (0.45 - 0.79)	53.6	0.42 (0.32 - 0.54)
Currently married	No	158 (2.8)	28.0		47.8		18.6		39.6		62.8	
	Yes	5526 (97.2)	35.6	1.42 (0.98 - 2.06)	56.7	1.43 (1.02 - 1.99)	29.6	1.84 (1.15 - 2.95)	39.8	1.01 (0.71 - 1.43)	68.6	1.30 (0.91 - 1.85)
Education	No formal	2484 (43.2)	25.1		43.3		18.6		25.4		56.5	
	Incomplete primary	1393 (25.1)	36.5	1.72 (1.44 - 2.06)	60.1	1.97 (1.67 - 2.33)	28.6	1.76 (1.42 - 2.17)	40.4	1.99 (1.68 - 2.37)	73.3	2.12 (1.73 - 2.59)
	Completed primary or above	1807 (31.7)	48.7	2.83 (2.37 - 3.39)	71.4	3.27 (2.74 - 3.92)	44.3	3.48 (2.85 - 4.25)	58.8	4.20 (3.53 - 5.01)	81.0	3.29 (2.67 - 4.05)
Literate	Illiterate	3195 (56.1)	26.0		46.3		21.3		27.9		60.5	
	Literate	2489 (43.9)	47.4	2.57 (2.23 - 2.95)	69.4	2.63 (2.25 - 3.06)	39.5	2.41 (2.00 - 2.90)	54.9	3.15 (2.72 - 3.66)	78.6	2.40 (2.03 - 2.84)
Occupation*	Unemployed	587 (10.6)	30.4		47.1		29.6		37.9		60.6	
	Manual	3833 (67.4)	32.6	1.10 (0.85 - 1.43)	53.7	1.30 (1.06 - 1.60)	25.4	0.81 (0.65 - 1.01)	35.4	0.90 (0.71 - 1.14)	66.6	1.30 (1.04 - 1.62)
	Professional	1263 (22.1)	46.7	2.00 (1.59 - 2.50)	69.4	2.55 (2.03 - 3.20)	41.0	1.65 (1.30 - 2.08)	53.9	1.92 (1.52 - 2.43)	77.9	2.29 (1.77 - 2.97)
Wealth	Most Poor	1026 (17.9)	24.7		43.7		18.8		25.2		57.0	
	2	1083 (19.2)	29.0	1.25 (0.97 - 1.60)	48.1	1.19 (0.96 - 1.48)	21.6	1.19 (0.95 - 1.50)	28.5	1.18 (0.92 - 1.51)	57.5	1.02 (0.79 - 1.31)
	3	1209 (21.2)	30.4	1.33 (1.05 - 1.69)	55.7	1.62 (1.28 - 2.04)	25.9	1.52 (1.19 - 1.93)	34.6	1.57 (1.21 - 2.03)	67.7	1.58 (1.23 - 2.03)
	4	1216 (21.3)	37.6	1.83 (1.42 - 2.37)	58.9	1.85 (1.45 - 2.35)	29.1	1.77 (1.35 - 2.34)	42.9	2.23 (1.69 - 2.93)	73.9	2.14 (1.65 - 2.77)
	Least Poor	1150 (20.4)	53.9	3.56 (2.64 - 4.79)	73.8	3.64 (2.79 - 4.75)	49.5	4.25 (3.17 - 5.69)	65.1	5.52 (4.22 - 7.23)	84.0	3.98 (2.87 - 5.50)
Religion	Other	565 (9.8)	37.0		60.8		34.6		47.9		73.3	
	Muslim	5119 (90.2)	35.3	0.93 (0.63 - 1.37)	56.0	0.82 (0.60 - 1.12)	28.7	0.76 (0.53 - 1.09)	38.9	0.69 (0.50 - 0.94)	68.0	0.77 (0.52 - 1.14)
Diabetic status*	Normoglycamic	4181 (74.0)	34.3		56.2		27.6		38.1		67.5	
	Intermediate hyperglycaemia	950 (17.0)	36.2	1.09 (0.91 - 1.29)	52.6	0.86 (0.73 - 1.03)	28.9	1.06 (0.88 - 1.28)	40.1	1.09 (0.90 - 1.31)	68.6	1.05 (0.90 - 1.23)
	Unknown diabetic	347 (6.3)	37.8	1.16 (0.87 - 1.55)	56.9	1.03 (0.76 - 1.38)	33.1	1.30 (0.96 - 1.76)	43.5	1.25 (0.95 - 1.64)	66.8	0.97 (0.70 - 1.33)
	Known diabetic	152 (2.6)	54.7	2.31 (1.60 - 3.34)	88.5	6.02 (3.64 - 9.95)	71.0	6.41 (4.32 - 9.51)	78.1	5.79 (3.86 - 8.70)	94.6	8.43 (4.15 - 17.11)
Total		5684 (100)	35.4		56.5		29.3		39.8		68.5	

*Note: missing diabetic status for 53 men; missing occupation data for 1 man.

Supplementary Table 3 Crude associations between <u>FEMALE</u> study population characteristics and ability to correctly report valid answers within five domains of diabetes knowledge. Proportions are cluster means and odds ratios and 95% confidence intervals are adjusted for the stratified, clustered survey design.

			Proportion able to report valid answers within each of the following domains of knowledge of diabetes:									
		Total	Causes		Symptoms		Complications		Prevention		Control	
		N (%)	%	OR (95% CI)	%	OR (95% CI)	%	OR (95% CI)	%	OR (95% CI)	%	OR (95% CI)
Age (years)	30-39	2376 (36.8)	34.9		58.4		28.2		40.1		67.3	
	40-49	1675 (26.0)	34.9	1.00 (0.88 - 1.14)	57.9	0.98 (0.85 - 1.12)	27.0	0.94 (0.82 - 1.08)	39.3	0.97 (0.85 - 1.11)	68.0	1.03 (0.90 - 1.18)
	50-59	1158 (17.8)	31.6	0.86 (0.74 - 1.00)	54.7	0.86 (0.74 - 1.00)	25.6	0.88 (0.74 - 1.04)	34.0	0.77 (0.66 - 0.90)	64.7	0.89 (0.75 - 1.06)
	60-69	878 (13.8)	25.8	0.65 (0.53 - 0.79)	41.0	0.49 (0.42 - 0.59)	21.3	0.69 (0.56 - 0.85)	26.2	0.53 (0.43 - 0.66)	52.8	0.54 (0.45 - 0.66)
	70+	369 (5.7)	12.4	0.27 (0.18 - 0.38)	26.9	0.26 (0.20 - 0.34)	9.6	0.27 (0.19 - 0.39)	15.3	0.27 (0.19 - 0.38)	42.7	0.36 (0.28 - 0.47)
Currently married	No	1350 (20.6)	24.8		44.2		20.8		29.0		57.5	
	Yes	5106 (79.4)	33.6	1.53 (1.28 - 1.83)	55.8	1.60 (1.37 - 1.86)	26.7	1.39 (1.17 - 1.64)	37.2	1.45 (1.23 - 1.72)	65.2	1.38 (1.17 - 1.63)
Education	No formal	3573 (55.0)	26.1		44.2		18.9		27.5		55.5	
	Incomplete primary	1384 (21.7)	37.7	1.72 (1.38 - 2.13)	63.5	2.20 (1.84 - 2.62)	28.7	1.73 (1.40 - 2.15)	41.2	1.85 (1.50 - 2.27)	72.9	2.15 (1.79 - 2.59)
	Completed primary or above	1499 (23.3)	39.8	1.88 (1.56 - 2.25)	66.1	2.46 (2.08 - 2.92)	38.0	2.63 (2.20 - 3.16)	49.1	2.54 (2.12 - 3.04)	74.1	2.29 (1.92 - 2.73)
Literate	Illiterate	4280 (66.1)	27.7		47.4		20.5		29.7		58.8	
	Literate	2176 (33.9)	39.7	1.72 (1.46 - 2.02)	65.2	2.08 (1.81 - 2.39)	35.1	2.10 (1.81 - 2.44)	46.8	2.09 (1.77 - 2.45)	73.1	1.91 (1.66 - 2.19)
Occupation*	Unemployed	6116 (94.7)	31.4		53.2		24.8		34.9		63.4	
	Manual	201 (3.1)	38.9	1.39 (0.89 - 2.15)	55.8	1.11 (0.79 - 1.56)	35.4	1.66 (1.07 - 2.57)	42.7	1.39 (0.91 - 2.11)	67.7	1.21 (0.85 - 1.72)
	Professional	138 (2.2)	37.1	1.29 (0.87 - 1.91)	62.1	1.44 (0.90 - 2.31)	41.2	2.13 (1.39 - 3.26)	49.9	1.86 (1.20 - 2.86)	68.0	1.23 (0.77 - 1.97)
Wealth	Most Poor	1405 (21.4)	21.8		41.3		18.9		25.6		53.9	
	2	1362 (21.4)	28.2	1.41 (1.13 - 1.76)	45.9	1.21 (1.02 - 1.44)	19.4	1.04 (0.82 - 1.31)	25.1	0.97 (0.79 - 1.20)	51.1	0.89 (0.69 - 1.15)
	3	1232 (19.2)	29.3	1.49 (1.21 - 1.83)	51.6	1.52 (1.24 - 1.85)	22.2	1.23 (0.96 - 1.57)	31.8	1.36 (1.09 - 1.68)	62.1	1.40 (1.12 - 1.75)
	4	1187 (18.1)	33.6	1.81 (1.41 - 2.33)	60.2	2.15 (1.68 - 2.75)	28.4	1.70 (1.29 - 2.25)	39.7	1.91 (1.48 - 2.47)	72.4	2.24 (1.77 - 2.83)
	Least Poor	1270 (19.9)	47.1	3.19 (2.45 - 4.17)	70.2	3.36 (2.52 - 4.48)	39.5	2.81 (2.12 - 3.70)	57.0	3.85 (2.96 - 5.01)	81.0	3.65 (2.76 - 4.83)
Religion	Other	575 (8.6)	29.3		49.2		29.1		40.7		66.7	
	Muslim	5881 (91.4)	32.0	1.14 (0.78 - 1.66)	53.9	1.21 (0.87 - 1.67)	25.1	0.82 (0.58 - 1.15)	35.0	0.78 (0.54 - 1.14)	63.3	0.86 (0.55 - 1.35)
Diabetic status*	Normoglycamic	4183 (65.0)	31.7		53.0		24.6		34.6		63.3	
	Intermediate hyperglycaemia	1505 (23.5)	30.9	0.97 (0.82 - 1.15)	51.5	0.94 (0.80 - 1.10)	23.5	0.95 (0.81 - 1.11)	35.2	1.03 (0.88 - 1.20)	61.6	0.93 (0.80 - 1.09)
	Unknown diabetic	571 (9.0)	31.3	0.98 (0.78 - 1.23)	52.0	0.96 (0.78 - 1.18)	25.5	1.05 (0.82 - 1.35)	34.0	0.98 (0.78 - 1.22)	61.6	0.93 (0.75 - 1.16)
	Known diabetic	158 (2.5)	44.4	1.72 (1.27 - 2.34)	85.9	5.38 (3.08 - 9.41)	66.0	5.95 (3.95 - 8.97)	70.0	4.42 (3.01 - 6.49)	94.5	9.96 (4.77 - 20.78)
Total		6456 (100)	31.8		53.5		65.5		35.5		63.2	

*Note: missing diabetic status for 40 women; missing occupation data for 1 woman.

Supplementary Table 4 Adjusted odds ratios showing associations between MALE study population characteristics and ability to correctly report valid answers within five domains of diabetes knowledge. Results are adjusted for all covariates and for the stratified, clustered survey design.

		Ability to report valid answers within each of the following domains of knowledge of diabetes:				
		Causes	Symptoms	Complications	Prevention	Control
		AOR 95% CI	AOR 95% CI	AOR 95% CI	AOR 95% CI	AOR 95% CI
Age	30-39					
	40-49	0.90 (0.76 - 1.07)	0.92 (0.78 - 1.08)	1.04 (0.87 - 1.23)	0.91 (0.77 - 1.07)	0.90 (0.77 - 1.07)
	50-59	0.96 (0.80 - 1.15)	1.00 (0.82 - 1.21)	1.14 (0.94 - 1.39)	0.90 (0.74 - 1.08)	0.95 (0.80 - 1.14)
	60-69	0.83 (0.68 - 1.01)	0.81 (0.66 - 1.00)	0.84 (0.67 - 1.06)	0.74 (0.60 - 0.92)	0.67 (0.54 - 0.83)
	70+	0.54 (0.37 - 0.78)	0.56 (0.42 - 0.77)	0.53 (0.36 - 0.78)	0.61 (0.43 - 0.86)	0.46 (0.34 - 0.62)
Currently married	No					
	Yes	1.27 (0.82 - 1.94)	1.20 (0.85 - 1.69)	1.78 (1.07 - 2.94)	0.92 (0.62 - 1.37)	1.02 (0.67 - 1.55)
Education	No formal					
	Incomplete primary	1.20 (0.95 - 1.52)	1.59 (1.25 - 2.01)	1.76 (1.29 - 2.40)	1.57 (1.26 - 1.96)	1.94 (1.49 - 2.53)
	Completed at least primary	1.22 (0.87 - 1.71)	1.86 (1.35 - 2.57)	2.94 (2.02 - 4.27)	2.14 (1.60 - 2.87)	2.49 (1.75 - 3.56)
Literate	Illiterate					
	Literate	1.68 (1.29 - 2.18)	1.25 (0.94 - 1.67)	0.80 (0.57 - 1.13)	1.25 (0.97 - 1.61)	0.86 (0.64 - 1.15)
Occupation	Unemployed					
	Manual	1.13 (0.85 - 1.50)	1.38 (1.09 - 1.73)	0.88 (0.67 - 1.14)	1.10 (0.85 - 1.44)	1.27 (0.97 - 1.67)
	Professional	1.34 (1.02 - 1.76)	1.76 (1.37 - 2.26)	1.09 (0.83 - 1.42)	1.32 (1.01 - 1.72)	1.43 (1.07 - 1.90)
Wealth	Most Poor					
	2	1.19 (0.92 - 1.54)	1.15 (0.92 - 1.43)	1.13 (0.89 - 1.42)	1.13 (0.88 - 1.44)	0.98 (0.76 - 1.25)
	3	1.15 (0.90 - 1.48)	1.42 (1.11 - 1.82)	1.31 (1.03 - 1.66)	1.34 (1.03 - 1.74)	1.44 (1.12 - 1.86)
	4	1.50 (1.15 - 1.97)	1.49 (1.16 - 1.92)	1.36 (1.03 - 1.81)	1.74 (1.30 - 2.33)	1.82 (1.40 - 2.36)
	Least Poor	2.52 (1.87 - 3.40)	2.49 (1.86 - 3.32)	2.69 (1.97 - 3.67)	3.50 (2.57 - 4.76)	3.02 (2.16 - 4.22)
Religion	Other					
	Muslim	1.09 (0.73 - 1.63)	0.94 (0.68 - 1.31)	0.93 (0.63 - 1.38)	0.83 (0.60 - 1.15)	0.88 (0.59 - 1.31)
Diabetic status	Normoglycamic					
	Intermediate hyperglycaemia	1.08 (0.90 - 1.30)	0.84 (0.70 - 1.02)	1.02 (0.84 - 1.24)	1.05 (0.86 - 1.29)	1.07 (0.90 - 1.26)
	Unknown diabetic	1.03 (0.75 - 1.42)	0.91 (0.67 - 1.25)	1.09 (0.80 - 1.48)	1.05 (0.78 - 1.41)	0.88 (0.63 - 1.23)
	Known diabetic	1.55 (1.06 - 2.28)	4.46 (2.60 - 7.65)	4.20 (2.70 - 6.52)	3.68 (2.36 - 5.74)	6.35 (3.06 - 13.18)

Note: n for multivariate analysis = 5630 due to missing occupation or diabetic status data for 54 men.

Supplementary Table 5 Adjusted odds ratios showing associations between FEMALE study population characteristics and ability to correctly report valid answers within five domains of diabetes knowledge. Results are adjusted for all covariates and for the stratified, clustered survey design.

		Ability to report valid answers within each of the following domains of knowledge of diabetes:				
		Causes	Symptoms	Complications	Prevention	Control
		AOR 95% CI	AOR 95% CI	AOR 95% CI	AOR 95% CI	AOR 95% CI
Age	30-39					
	40-49	1.04 (0.90 - 1.20)	1.07 (0.92 - 1.25)	1.08 (0.92 - 1.26)	1.01 (0.87 - 1.18)	1.08 (0.93 - 1.26)
	50-59	0.95 (0.81 - 1.13)	1.02 (0.86 - 1.22)	1.05 (0.84 - 1.32)	0.82 (0.69 - 0.99)	0.97 (0.80 - 1.18)
	60-69	0.77 (0.62 - 0.94)	0.61 (0.50 - 0.74)	0.91 (0.70 - 1.17)	0.58 (0.46 - 0.74)	0.60 (0.48 - 0.75)
	70+	0.31 (0.20 - 0.46)	0.28 (0.21 - 0.40)	0.34 (0.21 - 0.53)	0.26 (0.17 - 0.40)	0.35 (0.25 - 0.48)
Currently married	No					
	Yes	1.00 (0.82 - 1.22)	0.89 (0.74 - 1.07)	0.92 (0.76 - 1.11)	0.82 (0.67 - 1.00)	0.80 (0.66 - 0.97)
Education	No formal					
	Incomplete primary	1.24 (0.89 - 1.73)	1.74 (1.35 - 2.24)	1.47 (1.11 - 1.94)	1.36 (1.00 - 1.85)	1.86 (1.44 - 2.39)
	Completed at least primary	1.08 (0.70 - 1.66)	1.67 (1.17 - 2.39)	1.89 (1.34 - 2.67)	1.45 (0.96 - 2.18)	1.80 (1.23 - 2.63)
Literate	Illiterate					
	Literate	1.17 (0.79 - 1.74)	1.00 (0.73 - 1.37)	1.05 (0.78 - 1.41)	1.07 (0.73 - 1.56)	0.83 (0.61 - 1.15)
Occupation	Unemployed					
	Manual	1.59 (1.06 - 2.41)	1.23 (0.88 - 1.71)	1.91 (1.26 - 2.89)	1.60 (1.06 - 2.40)	1.33 (0.93 - 1.89)
	Professional	1.08 (0.73 - 1.59)	1.21 (0.78 - 1.86)	1.68 (1.13 - 2.51)	1.38 (0.91 - 2.11)	0.97 (0.62 - 1.50)
Wealth	Most Poor					
	2	1.37 (1.10 - 1.71)	1.14 (0.96 - 1.36)	0.99 (0.78 - 1.26)	0.95 (0.77 - 1.16)	0.86 (0.66 - 1.10)
	3	1.40 (1.14 - 1.73)	1.40 (1.12 - 1.74)	1.15 (0.89 - 1.49)	1.28 (1.02 - 1.62)	1.34 (1.06 - 1.69)
	4	1.70 (1.31 - 2.20)	1.93 (1.49 - 2.50)	1.50 (1.11 - 2.02)	1.75 (1.34 - 2.29)	2.07 (1.62 - 2.64)
	Least Poor	2.90 (2.20 - 3.83)	2.76 (2.03 - 3.77)	2.10 (1.55 - 2.85)	3.29 (2.47 - 4.40)	3.12 (2.32 - 4.18)
Religion	Other					
	Muslim	1.26 (0.85 - 1.86)	1.41 (0.99 - 2.01)	0.92 (0.64 - 1.33)	0.87 (0.59 - 1.28)	0.97 (0.61 - 1.54)
Diabetic status	Normoglycamic					
	Intermediate hyperglycaemia	0.98 (0.82 - 1.17)	0.96 (0.81 - 1.14)	0.96 (0.81 - 1.13)	1.04 (0.89 - 1.22)	0.94 (0.80 - 1.11)
	Unknown diabetic	0.96 (0.76 - 1.23)	0.98 (0.78 - 1.23)	1.07 (0.82 - 1.40)	0.97 (0.76 - 1.23)	0.94 (0.75 - 1.18)
	Known diabetic	1.60 (1.16 - 2.21)	5.76 (3.27 - 10.16)	6.11 (3.91 - 9.55)	4.52 (2.98 - 6.87)	10.23 (5.01 - 20.90)

Note: n for multivariate analysis = 6417 due to missing occupation or diabetic status data for 41 women.

Supplementary Table 6 Frequency, crude and adjusted odds ratios and 95% confidence intervals (CIs) for blood glucose testing and urine glucose testing among MEN by sociodemographic characteristic. Proportions are cluster means. All odds ratios and 95% CIs are adjusted for the stratified, clustered survey design. Adjusted odds ratios are adjusted for all covariates.

		Total	Ever Blood Glucose Test			Ever Urine Glucose Test		
		N	%	OR 95% CI	AOR 95% CI	%	OR 95% CI	AOR 95% CI
Age	30-39	1732 (30.0)	7.7			2.6		
	40-49	1376 (24.3)	11.4	1.54 (1.22 - 1.93)	1.56 (1.21 - 2.02)	4.4	1.74 (1.17 - 2.57)	1.55 (1.04 - 2.31)
	50-59	1135 (20.3)	17.7	2.58 (2.03 - 3.28)	2.90 (2.22 - 3.79)	6.6	2.66 (1.82 - 3.88)	2.29 (1.50 - 3.48)
	60-69	1039 (18.4)	21.1	3.20 (2.50 - 4.09)	3.02 (2.24 - 4.07)	8.8	3.62 (2.52 - 5.19)	2.55 (1.63 - 3.99)
	70+	402 (7.0)	19.5	2.91 (2.13 - 3.97)	1.93 (1.28 - 2.89)	7.7	3.15 (1.94 - 5.12)	2.02 (1.06 - 3.85)
Currently married	No	158 (2.8)	18.6			8.4		
	Yes	5526 (97.2)	13.8	0.70 (0.47 - 1.04)	0.79 (0.50 - 1.23)	5.3	0.60 (0.33 - 1.11)	0.68 (0.33 - 1.39)
Education	No formal	2484 (43.2)	7.3			3.0		
	Incomplete primary	1393 (25.1)	11.7	1.68 (1.31 - 2.16)	1.77 (1.23 - 2.54)	4.3	1.45 (0.97 - 2.15)	1.48 (0.91 - 2.42)
	Completed at least primary	1807 (31.7)	24.7	4.16 (3.34 - 5.18)	2.81 (1.78 - 4.43)	9.4	3.34 (2.54 - 4.39)	1.98 (1.17 - 3.37)
Literate	Illiterate	3195 (56.1)	8.0			3.2		
	Literate	2489 (43.9)	21.4	3.11 (2.59 - 3.73)	1.16 (0.81 - 1.67)	8.1	2.68 (2.11 - 3.41)	1.18 (0.75 - 1.87)
Occupation	Unemployed	587 (10.6)	29.8			12.1		
	Manual	3833 (67.4)	8.9	0.23 (0.18 - 0.30)	0.44 (0.31 - 0.61)	3.2	0.24 (0.17 - 0.33)	0.51 (0.32 - 0.79)
	Professional	1263 (22.1)	21.7	0.66 (0.51 - 0.84)	0.67 (0.47 - 0.96)	8.8	0.70 (0.51 - 0.96)	0.89 (0.56 - 1.41)
Wealth	Most Poor	1026 (17.9)	7.4			3.4		
	2	1083 (19.2)	6.5	0.87 (0.57 - 1.32)	0.74 (0.47 - 1.17)	3.1	0.91 (0.48 - 1.72)	0.83 (0.43 - 1.60)
	3	1209 (21.2)	11.2	1.59 (1.14 - 2.21)	1.22 (0.85 - 1.75)	4.3	1.31 (0.80 - 2.13)	1.02 (0.63 - 1.66)
	4	1216 (21.3)	12.7	1.82 (1.32 - 2.51)	1.17 (0.82 - 1.68)	4.7	1.41 (0.91 - 2.19)	0.91 (0.59 - 1.41)
	Least Poor	1150 (20.4)	30.7	5.58 (4.23 - 7.37)	2.40 (1.74 - 3.31)	11.0	3.54 (2.30 - 5.45)	1.19 (0.73 - 1.95)
Religion	Other	565 (9.8)	21.8			10.5		
	Muslim	5119 (90.2)	13.0	0.54 (0.41 - 0.70)	0.74 (0.55 - 0.99)	4.8	0.43 (0.28 - 0.66)	0.61 (0.38 - 0.96)
Diabetic status*	Normoglycamic	4181 (74.0)	10.9			3.5		
	Intermediate hyperglycaemia	950 (17.0)	12.9	1.21 (0.93 - 1.56)	0.95 (0.75 - 1.22)	4.6	1.33 (0.85 - 2.06)	1.08 (0.71 - 1.64)
	Unknown diabetic	347 (6.3)	16.4	1.59 (1.18 - 2.16)	1.02 (0.73 - 1.42)	5.7	1.64 (0.96 - 2.78)	1.14 (0.67 - 1.93)
	Known diabetic	152 (2.6)	99.0	832.20 (198.37 - 3,491.17)	548.51 (129.49 - 2,323.45)	60.6	41.99 (26.77 - 65.86)	21.59 (12.96 - 35.99)
TOTAL		5684 (100)	13.9			5.3		

Note: n for multivariate analysis = 5630 due to missing occupation or diabetic status data for 54 men.

Supplementary Table 7 Frequency, crude and adjusted odds ratios and 95% confidence intervals (CIs) for blood glucose testing and urine glucose testing among WOMEN by sociodemographic characteristic. Proportions are cluster means. All odds ratios and 95% CIs are adjusted for the stratified, clustered survey design. Adjusted odds ratios are adjusted for all covariates.

		Total	Ever Blood Glucose Test			Ever Urine Glucose Test		
		n (%)	%	OR 95% CI	AOR 95% CI	%	OR 95% CI	AOR 95% CI
Age	30-39	2376 (36.8)	11.3			4.2		
	40-49	1675 (26.0)	14.3	1.31 (1.10 - 1.55)	1.48 (1.20 - 1.82)	3.9	0.93 (0.70 - 1.24)	0.86 (0.60 - 1.24)
	50-59	1158 (17.8)	18.2	1.75 (1.42 - 2.15)	2.15 (1.67 - 2.77)	6.9	1.70 (1.20 - 2.40)	1.75 (1.14 - 2.68)
	60-69	878 (13.8)	16.0	1.50 (1.17 - 1.91)	1.87 (1.35 - 2.59)	6.8	1.66 (1.20 - 2.31)	1.91 (1.24 - 2.95)
	70+	369 (5.7)	13.1	1.19 (0.83 - 1.69)	1.20 (0.73 - 1.98)	5.1	1.22 (0.71 - 2.08)	1.16 (0.54 - 2.51)
Currently married	No	1350 (20.6)	16.2			5.6		
	Yes	5106 (79.4)	13.5	0.81 (0.66 - 0.98)	0.68 (0.54 - 0.87)	4.9	0.86 (0.63 - 1.16)	0.89 (0.59 - 1.35)
Education	No formal	3573 (55.0)	10.9			4.0		
	Incomplete primary	1384 (21.7)	14.0	1.33 (1.08 - 1.64)	1.44 (1.03 - 2.00)	5.3	1.35 (1.00 - 1.83)	1.88 (1.23 - 2.86)
	Completed at least primary	1499 (23.3)	21.5	2.23 (1.84 - 2.70)	2.10 (1.40 - 3.16)	7.2	1.86 (1.36 - 2.55)	2.39 (1.22 - 4.69)
Literate	Illiterate	4280 (66.1)	11.4			4.3		
	Literate	2176 (33.9)	19.2	1.85 (1.56 - 2.19)	1.15 (0.81 - 1.63)	6.4	1.53 (1.21 - 1.95)	0.83 (0.49 - 1.40)
Occupation	Unemployed	6116 (94.7)	13.9			5.1		
	Manual	201 (3.1)	13.6	0.98 (0.63 - 1.52)	1.22 (0.78 - 1.91)	2.2	0.41 (0.15 - 1.15)	0.56 (0.19 - 1.65)
	Professional	138 (2.2)	21.6	1.71 (1.12 - 2.60)	1.43 (0.90 - 2.25)	5.4	1.06 (0.52 - 2.18)	1.00 (0.40 - 2.50)
Wealth	Most Poor	1405 (21.4)	8.2			3.2		
	2	1362 (21.4)	8.1	0.99 (0.72 - 1.36)	0.95 (0.68 - 1.32)	3.7	1.15 (0.76 - 1.74)	1.06 (0.71 - 1.59)
	3	1232 (19.2)	10.9	1.38 (1.05 - 1.80)	1.38 (1.04 - 1.84)	4.5	1.43 (0.95 - 2.15)	1.35 (0.88 - 2.06)
	4	1187 (18.1)	16.1	2.16 (1.63 - 2.85)	2.09 (1.56 - 2.80)	5.0	1.58 (1.05 - 2.39)	1.39 (0.91 - 2.13)
	Least Poor	1270 (19.9)	27.9	4.33 (3.24 - 5.80)	3.39 (2.48 - 4.63)	8.9	2.93 (1.98 - 4.33)	1.82 (1.17 - 2.84)
Religion	Other	575 (8.6)	18.8			7.4		
	Muslim	5881 (91.4)	13.6	0.68 (0.48 - 0.96)	0.80 (0.54 - 1.18)	4.8	0.63 (0.36 - 1.11)	0.67 (0.38 - 1.20)
Diabetic status*	Normoglycamic	4183 (65.0)	11.0			3.3		
	Intermediate hyperglycaemia	1505 (23.5)	12.0	1.10 (0.90 - 1.36)	1.07 (0.87 - 1.33)	3.6	1.07 (0.75 - 1.54)	1.07 (0.74 - 1.55)
	Unknown diabetic	571 (9.0)	17.9	1.76 (1.34 - 2.32)	1.61 (1.22 - 2.12)	6.2	1.91 (1.26 - 2.90)	1.83 (1.19 - 2.83)
	Known diabetic	158 (2.5)	97.1	266.91 (92.78 - 767.87)	298.07 (95.72 - 928.14)	58.6	41.19 (26.66 - 63.66)	37.57 (23.63 - 59.73)
TOTAL		6456 (100)	14.1			5.0		

Note: n for multivariate analysis = 6417 due to missing occupation or diabetic status data for 41 women.
